# Determination of the Exchange Current Density at Lithium │ Polymer Electrolyte Interfaces

**DOI:** 10.1002/advs.202514492

**Published:** 2025-11-27

**Authors:** Katrin Geng, Bryce A. Tappan, Stefano Passerini, Yang Shao‐Horn, Dominic Bresser

**Affiliations:** ^1^ Helmholtz Institute Ulm (HIU) 89081 Ulm Germany; ^2^ Karlsruhe Institute of Technology (KIT) 76131 Karlsruhe Germany; ^3^ Research Laboratory of Electronics Massachusetts Institute of Technology (MIT) Cambridge MA 02139 USA; ^4^ Austrian Institute of Technology (AIT) Center for Transportation Technologies Vienna 1210 Austria; ^5^ Department of Materials Science and Engineering Massachusetts Institute of Technology (MIT) Cambridge MA 02139 USA; ^6^ Department of Mechanical Engineering Massachusetts Institute of Technology (MIT) Cambridge MA 02139 USA; ^7^ Ulm University (UUlm) 89069 Ulm Germany

**Keywords:** battery, charge transfer resistance, exchange current density, lithium metal, polymer electrolyte

## Abstract

While interfacial processes can dominate the internal resistance in solid‐state batteries, the (electro‐)chemical reactions occurring at the lithium│polymer interface are complex and dynamic upon cycling. A central factor for evaluating such reactions is the exchange current density *j*_0_ that characterizes the kinetics of the interfacial charge transfer. However, its determination is challenging due to superimposed impedance contributions from the solid electrolyte interphase and interfacial charge transfer reactions. Moreover, different methodologies for determining *j*_0_ can lead to different *j*_0_ values. Herein, a carefully validated method to determine *j*_0_ for polymer electrolytes is reported, using the example of polyethylene oxide‐based systems, by combining electrochemical impedance spectroscopy, Bayesian inference analysis, and distribution of relaxation times analysis. These impedance‐based methodologies are validated via the determination of *j*_0_ using DC polarization measurements that are fit to a modified Butler–Volmer model, enabling the reliable determination of *j*_0_ for polymer electrolytes and, thus, the analysis of the interfacial processes and reactions occurring in such systems.

## Graphical abstract

This work presents a comprehensively validated method to determine the exchange current density (*j*_0_) at the interface of lithium metal and polymer electrolytes by combining electrochemical impedance spectroscopy with Bayesian inference and distribution of relaxation times analyses to successfully deconvolute solid electrolyte interphase and charge transfer contributions, as cross‐validated in parallel in two laboratories.

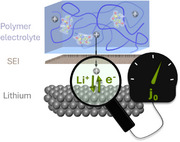

## 1 Introduction

The ubiquity of portable electronic devices and the increasing share of electric vehicles illustrate the growing demand for high‐energy‐density batteries. One of the most promising approaches to achieve energy densities beyond those provided by the well‐established lithium‐ion technology is the transition to lithium‐metal negative electrodes, owing to their low electrochemical potential (−3.04 V vs the standard hydrogen electrode) and high theoretical specific capacity (3860 mAh g^−1^).^[^ ^1^, ^2^ ^]^ However, several challenges remain toward this highly desirable goal, including the formation of dendritic lithium deposits, excessive parasitic side reactions with conventional liquid electrolytes, and the resulting severe safety concerns.^[^ ^1^, ^3^, ^4^ ^]^ Electrolytes based on polyethylene oxide (PEO) are the only polymer electrolytes that have already been successfully commercialized, benefitting from low cost, ease of processing, and the ability to establish a good interfacial contact at the electrode│electrolyte interfaces.^[^ ^5^, ^6^ ^]^ Nonetheless, several challenges remain. Besides the relatively low ionic conductivity at ambient temperatures and limited stability towards oxidation of PEO‐based electrolytes, lithium metal reacts with commonly employed conducting lithium salts and also with PEO to produce passivating films (commonly referred to as solid electrolyte interphase, SEI) composed of salt decomposition products like LiF,^[^ ^7^ ^]^ Li_2_CO_3_,^[^ ^7^ ^]^ Li_3_N^[^ ^7^ ^]^ as well as lithium alkoxides^[^ ^8^, ^9^ ^]^ and Li_2_O.^[^ ^7^, ^8^ ^]^ While a passivating SEI film can limit further reduction of the polymer and conducting salt with lithium metal, the reactivity at the polymer│Li interface reduces the capacity during cycling, as it decreases the amount of metallic lithium available in the cell, and the increasing anode resistance leads to an incomplete charge and discharge of the cell.^[^ ^10^ ^]^ Thus, to fully realize the potential of lithium‐metal electrodes, a better understanding of the reactions occurring at the electrode│electrolyte interface, including the charge transfer reaction, is imperative. One common metric by which the kinetics of charge transfer reactions are measured is the exchange current density *j*_0_. The exchange current density describes the magnitude of the forward and reverse reaction of an electrochemical process at equilibrium.^[^ ^11^, ^12^ ^]^ A high *j*_0_, for instance, indicates that a given electrochemical reaction can be driven at a fast rate with a low overpotential.^[^ ^13^ ^]^ Moreover, an accurate determination of using *j*_0_ as a measure for estimating and ideally forecasting the plating and stripping efficiency in lithium‐metal batteries.^[^ ^12^, ^14^ ^]^

Despite its importance, however, the determination of *j*_0_ is frequently far from simple. In fact, recent works have shown that reported exchange current density values for lithium metal stripping and plating in similar systems vary by several orders of magnitude between different methods and research groups.^[^ ^14^, ^15^ ^]^ For liquid electrolytes, the exchange current density is traditionally determined with a rotating disc electrode.^[^ ^16^, ^17^ ^]^ For polymer (or generally solid) electrolytes, however, rotating disc electrodes and other more advanced techniques, such as transient voltammetry with ultramicroelectrodes,^[^ ^18^ ^]^ are either impossible or extremely difficult to implement experimentally,^[^ ^19^ ^]^ rendering the exchange current density determination substantially more challenging. Additionally, linear fitting of Tafel plots can yield inaccurate results.^[^ ^17^ ^]^ Other approaches reported so far include less straightforward pulse techniques,^[^ ^20^, ^21^ ^]^ as well as the galvanostatic and potentiostatic intermittent titration technique (GITT, PITT),^[^ ^15^ ^]^ and a galvanostatic technique recently reported for Li│PEO│Li cells.^[^ ^22^, ^23^ ^]^ While the latter appears rather easy to use for polymer electrolytes, it does not clearly distinguish the charge transfer resistance *R*_ct_ from the SEI resistance, leading to an overestimation of *R*_ct_.^[^ ^22^ ^]^ An alternative method suitable for polymer electrolytes is electrochemical impedance spectroscopy (EIS). By EIS, the charge transfer resistance can be determined and directly converted into the exchange current density via the edge‐case of the Butler–Volmer equation at small overpotentials, Equation (1):

(1) $$j_{0}=\frac{\mathrm{RT}}{\mathrm{zF}R_{\mathrm{ct}}}$$

with *R* being the ideal gas constant, *T* the temperature in *K*, *z* the number of electrons transferred (in our case equal to 1), *F* the Faraday constant, and *R*_ct_ the charge transfer resistance.^[^ ^11^ ^]^ Since EIS enables the separation of signals arising from electrochemical processes that occur on different time scales, it can isolate the charge transfer resistance from other sources of resistance in batteries (electrolyte resistance, resistance from diffusional processes, etc.), which is an advantage over GITT or PITT.^[^ ^15^, ^24^ ^]^ For this reason, EIS has provided the most reliable exchange current density values compared to GITT and PITT for Li││LiNi_0.4_Mn_0.3_Co_0.3_O_2_ cell employing a liquid electrolyte.^[^ ^15^ ^]^ Moreover, EIS is nondestructive, easily applicable, and provides rich information about the interfaces in batteries. Nevertheless, the data analysis of EIS is not straightforward, since the conventional way of EIS spectra analysis—equivalent circuit model fitting—requires a suitable model to yield meaningful results. For Li│PEO│Li cells, a wide variety of different equivalent circuit models has been employed in the literature, consisting of resistors (R), constant phase elements (CPEs), expressing nonideal behavior of a capacitor together with the frequency independent constant *n* between 0 (CPE = resistor) and 1 (CPE = ideal capacitor), and Warburg elements (W) in various combinations.^[^ ^25^, ^26^, ^27^, ^28^, ^29^, ^30^, ^31^ ^]^ Generally speaking, one, two, or three (R)(CPE)‐elements are used in the literature, nested or in series, to fit the interphase‐related semicircle in the medium frequency range of EIS spectra recorded for Li│polymer│Li cells.^[^ ^26^, ^27^, ^28^, ^29^, ^30^, ^31^ ^]^ Some studies use a single (R)(CPE)‐element, interpreted as charge transfer or interphase (SEI) resistance while others use two and more (R)(CPE)‐elements, assigned to charge transfer and interphase resistance,^[^ ^26^, ^27^, ^28^, ^29^, ^30^, ^31^ ^]^ although the physicochemical interpretation of the additional contribution(s) can remain ambiguous.^[^ ^26^, ^31^ ^]^ For example, the mid‐frequency region in an EIS spectrum of a Li│polymer│Li cell, often just referred to as “interphase resistance” or “charge transfer resistance”^[^ ^25^, ^32^, ^33^, ^34^, ^35^ ^]^ has been reported to contain (partially) overlapping contributions from the SEI and *R*_ct_, which complicates the *j*_0_ extraction.^[^ ^27^, ^36^, ^37^ ^]^ Therefore, researchers are confronted with the question of which equivalent circuit is the most suitable for the accurate extraction of *R*_ct_ and determination of *j*_0_ by EIS (Equation (1)).

Herein, we approach the aforementioned challenge to identify the most appropriate equivalent circuit model for fitting EIS data and to disentangle the charge transfer resistance contributions from the contribution of the surface layer by employing a comprehensive set of methodologies, using PEO as well‐established state‐of‐the‐art polymer electrolyte. Specifically, we supplement traditional EIS equivalent circuit model fitting with complementary techniques, including Bayesian inference analysis (AutoEIS), distribution of relaxation times (DRT) analysis, and extended cycling experiments that provide insights into the distinct temporal evolution of the SEI resistances and *R*_ct_. Moreover, we apply cyclic voltammetry coupled with fitting of the data to a modified Butler–Volmer model to corroborate the EIS‐determined exchange current density values, thus, offering a comprehensive, conclusive, and generally applicable method for accurately determining *j*_0_ in LMBs with polymer electrolytes.

## 2 Results and Discussion

### 2.1 Identifying a Suitable Equivalent Circuit Model for EIS Fitting

To find the most suitable equivalent circuit model to extract the charge transfer resistance, EIS data of the well‐established polymer electrolyte system consisting of PEO and lithium bis(trifluoromethanesulfonyl)imide (LiTFSI) were subjected to three complementary analyses: (1) conventional equivalent circuit fitting, (2) Bayesian inference analysis of EIS data using the AutoEIS tool, and (3) DRT analysis.

**Figure 1:**
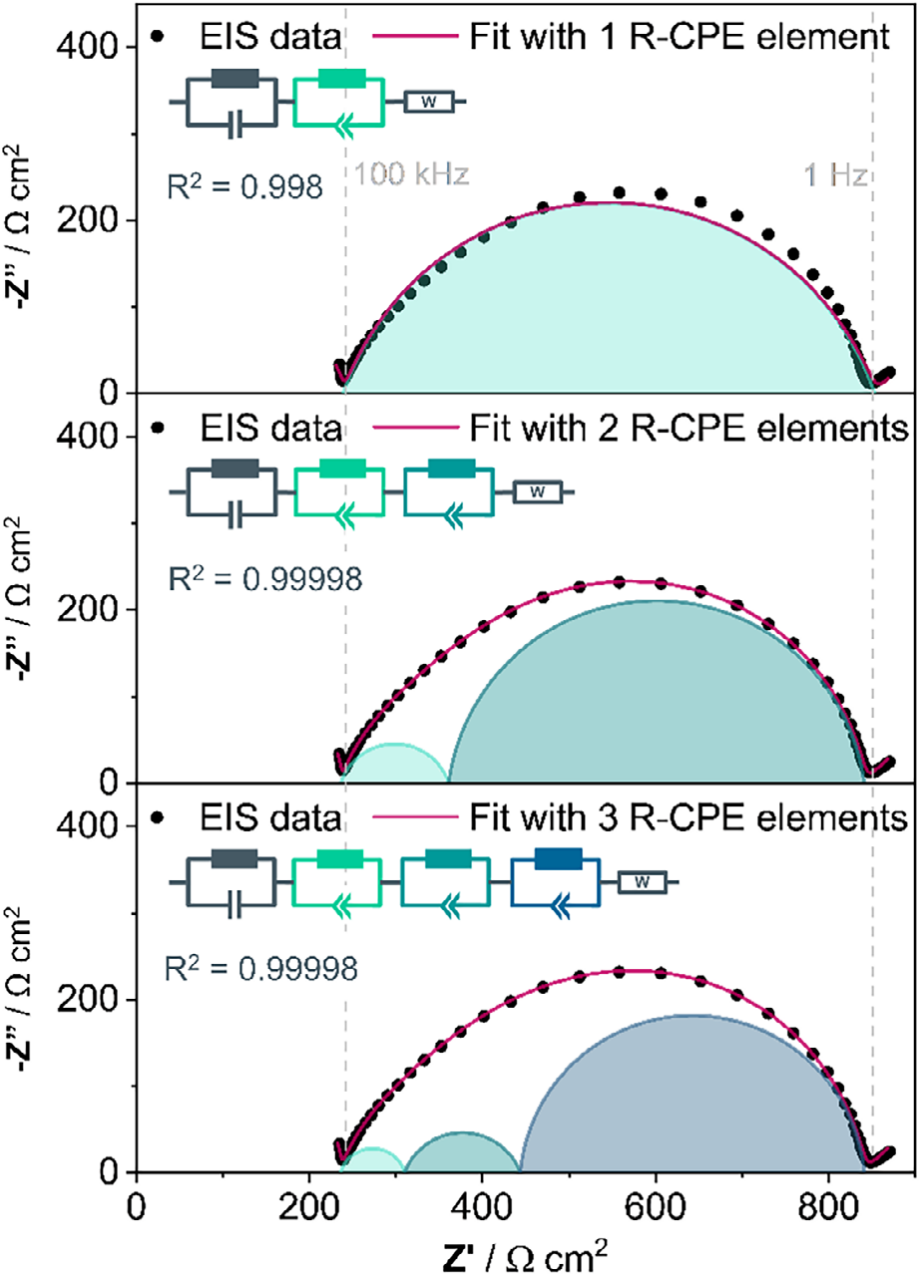
Conventional equivalent circuit model fitting of an impedance spectrum recorded for a Li│PEO+LiTFSI│Li cell with three different equivalent circuit models. The equivalent circuit models used for fitting are displayed in each subgraph: the mid‐frequency region (from 100 kHz to 1 Hz) was fitted with one, two, or three R‐CPE elements (from top to bottom). The impedance spectrum was recorded at 40 °C after 6 h of rest.

**Figures** **1** and S1 (Supporting Information) illustrate the results of conventional equivalent circuit model fitting with three different equivalent circuit models. We began the fitting procedure with the simplest equivalent circuit model that we considered potentially appropriate for fitting the data. The high‐frequency region (≥1 MHz – 100 kHz) consists of the response from ionic conductivity in the bulk electrolyte as well as the electrical resistance from current collectors and cables,^[^ ^26^, ^38^ ^]^ and can be fitted with a simple resistor R or (R)(C)‐element. This resistance corresponds to the *x*‑axis intercept of the impedance spectrum at high frequencies (compare Figure 1 and Figure S1, Supporting Information).^[^ ^38^ ^]^ For low frequencies (<1 Hz), a Warburg element W was used to fit the diffusion due to ionic transport in the electrolyte (for a lower frequency cut‐off, Warburg short behavior would be observed, not shown here).^[^ ^26^ ^]^ The slightly depressed semicircular feature in the intermediate region (100 kHz to 1 Hz), containing the overall response of the Li│PEO interphase, was initially fitted with one (R)(CPE)‐element, which yielded significant deviation between the data and the fit (refer to the upper panel in Figure 1).

In agreement with Bouchet et al.,^[^ ^26^ ^]^ we may consider the phenomena taking place in our system, as reflected by the impedance response (ion transport in the electrolyte, ion transport through the SEI, and charge transfer), to occur in series. The observed depressed semicircular feature in the Nyquist plot is an indication that multiple electrochemical phenomena are operating on roughly similar time scales.^[^ ^26^ ^]^ Therefore, such a depressed semicircular response can be fitted with several (R)(CPE)‐elements in series with (slightly) different time constants (or angular frequencies).^[^ ^26^ ^]^ The inclusion of one more (R)(CPE)‐element in series to the equivalent circuit model remarkably improves the fit as shown in the middle panel in Figure 1, matching the experimental data very well. Using three (R)(CPE)‐elements in series in the model for the mid‐frequency region, the fit agrees well with the experimental data points (Figure 1 bottom panel). However, not all the component values in the equivalent circuit model could be determined with high accuracy by the fitting software, indicating overfitting (compare Table S1, Supporting Information). Furthermore, the best models are commonly those that achieve satisfactory fits with the fewest possible parameters while still maintaining physical and chemical interpretability.^[^ ^38^ ^]^ Thus, the conventional equivalent circuit fitting method indicates that two (R)(CPE)‐elements represent the optimal model for fitting the mid‐frequency “interphase” region of EIS data from Li│PEO│Li cells. This observation – that one (R)(CPE)‐element is insufficient for fitting the EIS data, that two (R)(CPE)_‐_elements result in a satisfactory fit, and that three (R)(CPE)‐elements do not improve the fit but instead seem to represent overfitting – holds true for different temperatures (40 °C vs 80 °C, compare Figure 1 and Figure S1a, Supporting Information), and was confirmed in multiple measurements (Figure S1b, Supporting Information).

**Figure 2:**
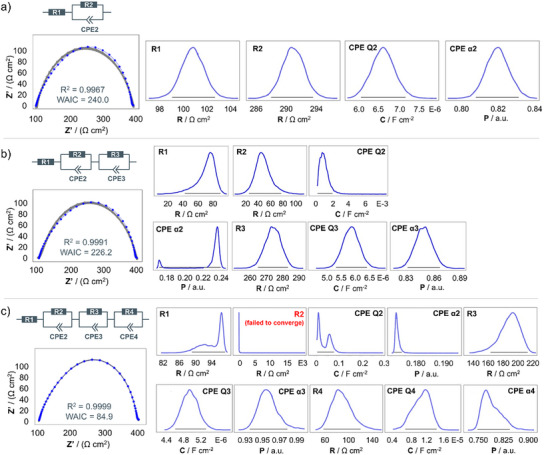
Bayesian inference analysis performed on an EIS spectrum in the mid‐frequency range (194 kHz to 1 Hz) using AutoEIS. The impedance spectrum was recorded for a Li│PEO+LiTFSI│Li cell at 40 °C after 6 h of rest. Results are shown for three different equivalent circuit models: a) R‐(R)(CPE), b) R‐(R)(CPE)‐(R)(CPE), and c) R‐(R)(CPE)‐(R)(CPE) (from top to bottom). For each of these, predictive Nyquist plots are shown (left), together with the respective probability distributions for the different equivalent circuit model components.

As conventional equivalent circuit model fitting can be susceptible to user bias and often results in nonunique solutions,^[^ ^39^, ^40^ ^]^ we employed a tool developed by Zhang et al.,^[^ ^40^ ^]^ called “AutoEIS,” that can optimize equivalent circuit models by using Bayesian inference statistics. When fitting EIS data, AutoEIS generates probability distributions for each equivalent circuit model component.^[^ ^40^ ^]^ These posterior distributions, together with other parameters like the inference quality and predictive plots, serve as the basis for the evaluation of the model plausibility.^[^ ^40^ ^]^ Ranking the models according to their widely applicable information criterion (WAIC) ensures a preference for simpler equivalent circuit models with sufficient fitting quality.^[^ ^40^ ^]^ By applying AutoEIS to our data, we found similar tendencies to those observed with conventional equivalent circuit fitting. The results for three different equivalent circuit models are presented in **Figure** **2**a–c and Figure S2 (Supporting Information). Both with one and with two (R)‐(CPE)‐elements for fitting the 194 kHz to 1 Hz frequency region, the resulting probability distributions for the circuit elements show a Gaussian shape, which indicates that they are well‐behaved. When comparing the respective predictive Nyquist plots (left panel in Figure 2a,b) together with the *R* ^2^ and WAIC values, the fit improved with two (R)(CPE)‐elements instead of one. The use of a model with three (R)(CPE)‐elements^[^ ^40^ ^]^ (Figure 2c) clearly shows that the probability distribution for certain circuit elements (R^2^) failed to converge, indicating and confirming the overfitting discussed earlier.

**Figure 3:**
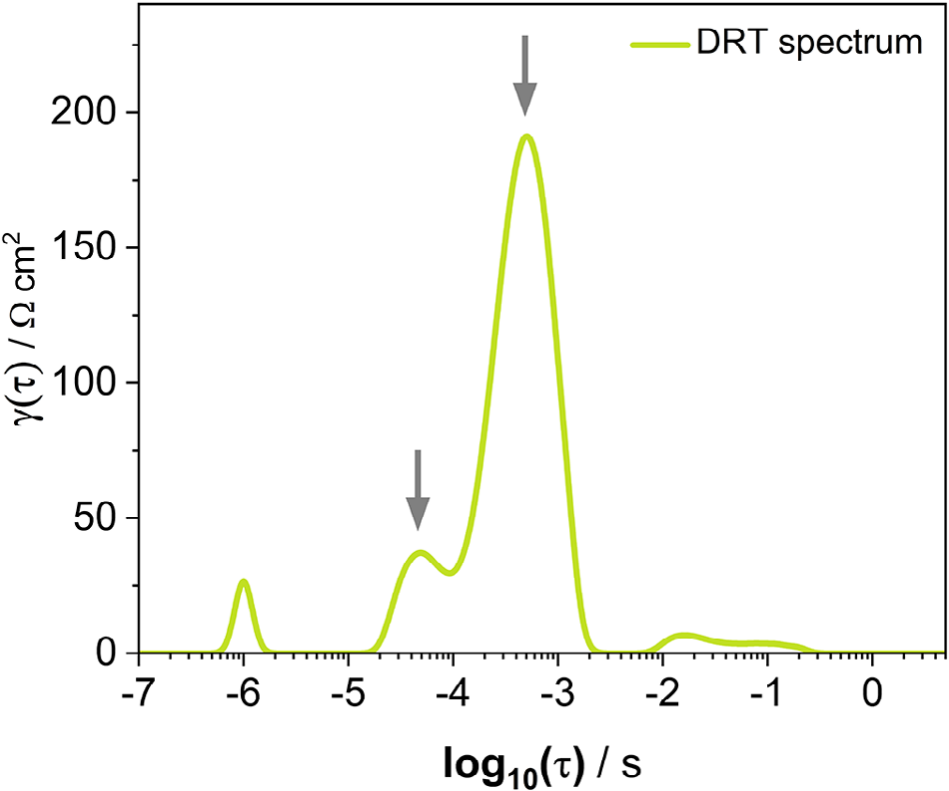
Distribution of relaxation times (DRT) analysis of an impedance spectrum. EIS was performed from 1 MHz to 5 Hz for a Li│PEO+LiTFSI│Li cell at 40 °C after 24 h of equilibration time. The DRT spectrum shows two major peaks in the mid‐frequency region between 10^−5^ and 10^−3^ s.

As a third approach to find the most suitable equivalent circuit model, DRT was performed, where frequency‐based impedance measurements are converted into the time domain,^[^ ^41^, ^42^ ^]^ allowing for the separation of contributions to EIS happening on different time scales.^[^ ^24^ ^]^ One of the main advantages of DRT is that classical pitfalls of equivalent circuit model fitting, where the user has to decide on the number and nature of processes to choose for the model prior to fitting, can be circumvented because its representation is “model‐free,” while each peak in the DRT spectrum corresponds to a physicochemical process.^[^ ^24^, ^43^, ^44^ ^]^ The integrated area under the respective DRT peak (with ln(τ) on the *x*‑axis) represents the corresponding resistance of the process and can be obtained through a Gaussian fit. The results of the DRT analysis are presented in **Figure** **3**. Apart from a feature at high frequencies (≈10^−6^ s), which can be assigned to the bulk electrolyte,^[^ ^36^, ^44^ ^]^ there are two main peaks visible, i.e., a smaller peak at ≈10^−4^ s and a larger peak at ≈10^−3^ s. In Figure S3 (Supporting Information), the DRT results from our two different labs are shown. Both independent DRT analyses yielded very similar results. In both cases, despite slightly different experimental conditions, two peaks are visible in the mid‐frequency region of the spectra. Hence, the DRT analysis also points towards using two (R)(CPE)‐elements for fitting the mid‐frequency region of the EIS data. Consequently, R_1_‐(R_2_)(CPE_2_)‐(R_3_)(CPE_3_)‐W (with R_1_ being R or (R)(C) or L‐R) could be deduced as suitable equivalent circuit model for fitting Li│PEO│Li EIS data, based on conventional equivalent circuit model fitting, AutoEIS based on Bayesian inference analysis, and DRT. This equivalent circuit has also been applied, e.g., by Munichandraiah et al.,^[^ ^36^ ^]^ Tappan et al.,^[^ ^45^ ^]^ and Appetecchi et al.^[^ ^46^ ^]^ for similar systems.

In summary, the combined results from conventional fitting, AutoEIS, and DRT consistently support a two‐process interfacial model. While the model provides an excellent fit across techniques and experimental conditions, the underlying physicochemical nature of these processes requires further interpretation. Therefore, we next turn to a detailed analysis of the physical and chemical origin of the impedance contributions to assign them to specific interfacial phenomena.

### 2.2 Physicochemical Interpretation of the Equivalent Circuit Model

**Figure 4:**
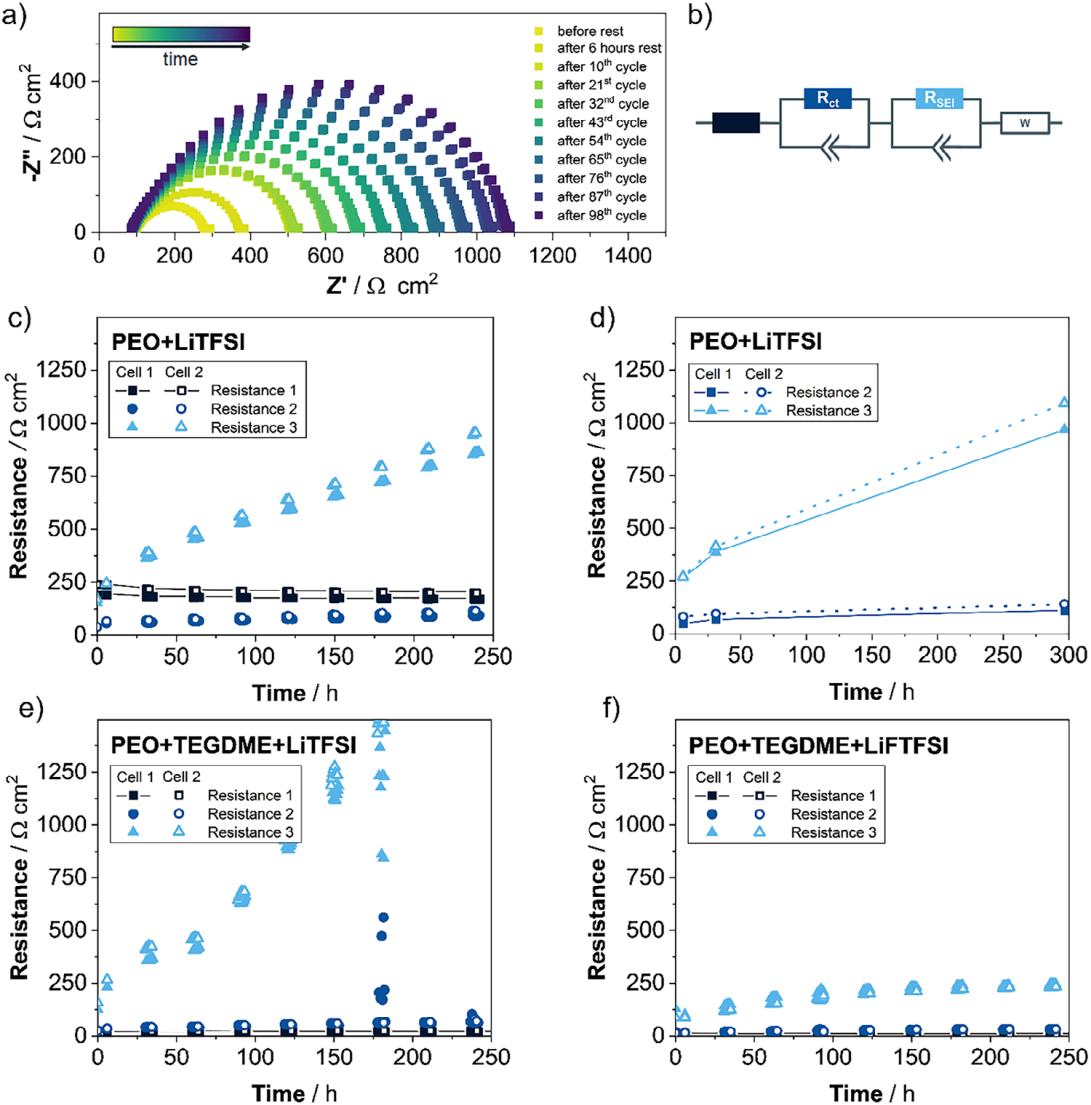
Electrochemical impedance spectroscopy over the course of cycling with different polymer electrolytes. a) Impedance spectra recorded for a Li|PEO+LiTFSI|Li cell at 40 °C in the frequency range from 100 mHz to 1 MHz between different plating/stripping cycles. Plating/stripping was performed with a current density of 0.1 mA cm^−2^ and an areal capacity of 0.1 mAh cm^−2^ per half‐cycle. b) The equivalent circuit model used for fitting the EIS data in panel (a). c) Resistance values obtained by fitting the impedance spectra during plating/stripping of two Li│PEO+LiTFSI│Li cells (refer to panel (a)) with the equivalent circuit model displayed in panel (b). Resistance 2 (*R*_ct_) and Resistance 3 (*R*_SEI_) were divided by 2 to obtain “per‐interface” values. d) Resistance values obtained via Bayesian inference analysis with AutoEIS, which was applied to selected impedance spectra during plating/stripping of two Li│PEO+LiTFSI│Li cells (refer to panels (a)–(c)). e) Resistance values obtained by fitting the impedance spectra recorded for a Li│PEO+TEGDME+LiTFSI│Li cell at 40 °C between different plating/stripping cycles with a current density of 0.1 mA cm^−2^ and an areal capacity of 0.1 mAh cm^‑2^ per half‐cycle, using the model shown in panel (b). f) Resistance values obtained by fitting the impedance spectra recorded for a Li│PEO+TEGDME+LiFTFSI│Li cell at 40 °C between different plating/stripping cycles with a current density of 0.1 mA cm^−2^ and an areal capacity of 0.1 mAh cm^−2^ per half‐cycle using to the model shown in panel (b).

Equivalent circuit modeling, AutoEIS, and DRT analysis all indicate that the impedance at the Li│PEO interface arises from two primary electrochemical processes, where likely the first is the charge transfer, and the second is the ion transport through the rather resistive SEI film, consisting of electrolyte decomposition products such as LiF, Li_2_CO_3_, Li_3_N, lithium alkoxides, and/or Li_2_O.^[^ ^7^, ^8^, ^9^ ^]^ However, through EIS measurements alone, it is not immediately clear which impedance response corresponds to the charge transfer and which one to the ion transport in the SEI film. In order to assign the impedance responses to these physicochemical processes, galvanostatic Li stripping and plating was performed on symmetric Li│PEO│Li cells. EIS spectra were recorded after every 10 plating/stripping cycles at OCV conditions. **Figure** **4**a shows the evolution of the impedance over time for such a Li│PEO│Li cell. Resistance values were obtained by fitting this EIS data to the previously confirmed equivalent circuit model R_1_‑(R_2_)(CPE_2_)‑(R_3_)(CPE_3_)‑W (see Figure 4b), and by dividing the resistance values R_2_ and R_3_ by two to yield resistance values per electrode. These resistances, determined over the course of cycling, are presented in Figure 4c–f. Figure 4c shows one constant resistance (Resistance 1), which is the resistor in series that corresponds to the bulk ionic resistance of the electrolyte and other electronic resistances of the system including cables, current collectors etc.^[^ ^26^, ^47^ ^]^ The other two resistors come from the (R_2_)(CPE_2_)‑(R_3_)(CPE_3_) circuit elements that capture the interfacial response (i.e., the mid‐frequency semicircle of the Nyquist plot in Figure 4a). Out of these two resistors, one increases slightly over the course of 250 h, while the other one increases substantially. Since the SEI has been shown to be continuously growing,^[^ ^48^, ^49^ ^]^ the substantially increasing resistance (Resistance 3) may be assigned to the lithium ion transport through a growing SEI layer. Thus, the remaining resistor of the equivalent circuit model (Resistance 2) may be assigned to the charge transfer step (in agreement with previous work^[^ ^18^, ^44^, ^46^ ^]^).

In addition to the resistance values, the CPE values obtained from EIS fitting can be examined and converted to a more meaningful capacitance value, the equivalent capacitance, that results in the same time constant via Equation (2).^[^ ^26^, ^50^ ^]^

(2) $$C_{equiv}=R^{\frac{\left(1-n\right)}{n}}CPE^{\frac{1}{n}}\;$$

For the two mid‐frequency EIS contributions (the charge transfer and charge transport through the SEI), the equivalent capacitances, calculated from the CPE values with Equation (2) at 40 °C, are *C*_equiv_(CT) = 1.0 µF cm^−2^ and *C*_equiv_(SEI) = 1.6 µF cm^−2^. The electrode interfacial capacitance values reported by Peled^[^ ^49^ ^]^ for different alkali or alkaline earth metal battery systems are in the small single‐digit µF cm^−2^ range, thereby agreeing well with our findings. When using the *R*, CPE, and *n* values published by Munichandraiah et al.^[^ ^36^ ^]^ for Li│PEO+LiClO_4_+PC│Li at ambient temperature, one obtains *C*_equiv_(CT) = 0.97 µF cm^−2^ and *C*_equiv_(SEI) = 1.4 µF cm^−2^, which are also in excellent agreement with our findings for the Li│PEO+LiTFSI│Li system. Selected data (after 6 h of rest, after 10 cycles and after more than 100 cycles) were also fitted using AutoEIS (Figure 4d), which revealed a good agreement between the conventional equivalent circuit fitting and the Bayesian inference analysis (compare Figure 4c,d).

To validate the fit model and its assignment to the physicochemical processes occurring, we performed the same cycling experiment also with different electrolyte compositions. For this purpose, in a first step, tetraethylene glycol dimethyl ether (TEGDME) was added to the previously used PEO+LiTFSI solid polymer electrolyte. In addition, in a second step, we investigated such a PEO‐based electrolyte containing another conducting salt (i.e., lithium (fluorosulfonyl)(trifluoromethanesulfonyl)imide, LiFTFSI). As evident from Figure 4c–f, for all electrolyte compositions, duplicate cells generally yielded similar results, indicative of experimental reproducibility. With the addition of TEGDME to the earlier used PEO+LiTFSI mixture (Figure 4e), the rise of the SEI resistance becomes much more pronounced (Figure 4c,e). In fact, when adding liquid TEGDME—in its role as plasticizing agent—the segmental dynamics and ion mobility increase,^[^ ^51^, ^52^ ^]^ and, therefore, also the side reactions at the interface. In comparison, with a different conducting salt (i.e., LiFTFSI instead of LiTFSI), the SEI layer growth shows a much lower increase (Figure 4e,f), which may be explained by earlier findings that LiFTFSI yields a much more stable SEI due to facile bond breaking between S and F, resulting in an inorganic‐rich (especially LiF‐rich) and thinner SEI layer.^[^ ^53^, ^54^, ^55^ ^]^ Such inorganic‐rich layer is commonly beneficial for passivating the lithium‐metal electrode,^[^ ^55^, ^56^ ^]^ leading to a relatively less pronounced growth of the SEI layer (Figure 4f). Consequently, the distinct SEI layer increase expected for the different electrolyte systems PEO+LiTFSI, PEO+TEGDME+LiTFSI, and PEO+TEGDME+LiFTFSI confirms the assignment of Resistance 3 to the SEI resistance and Resistance 2 to the charge transfer resistance.

**Figure 5:**
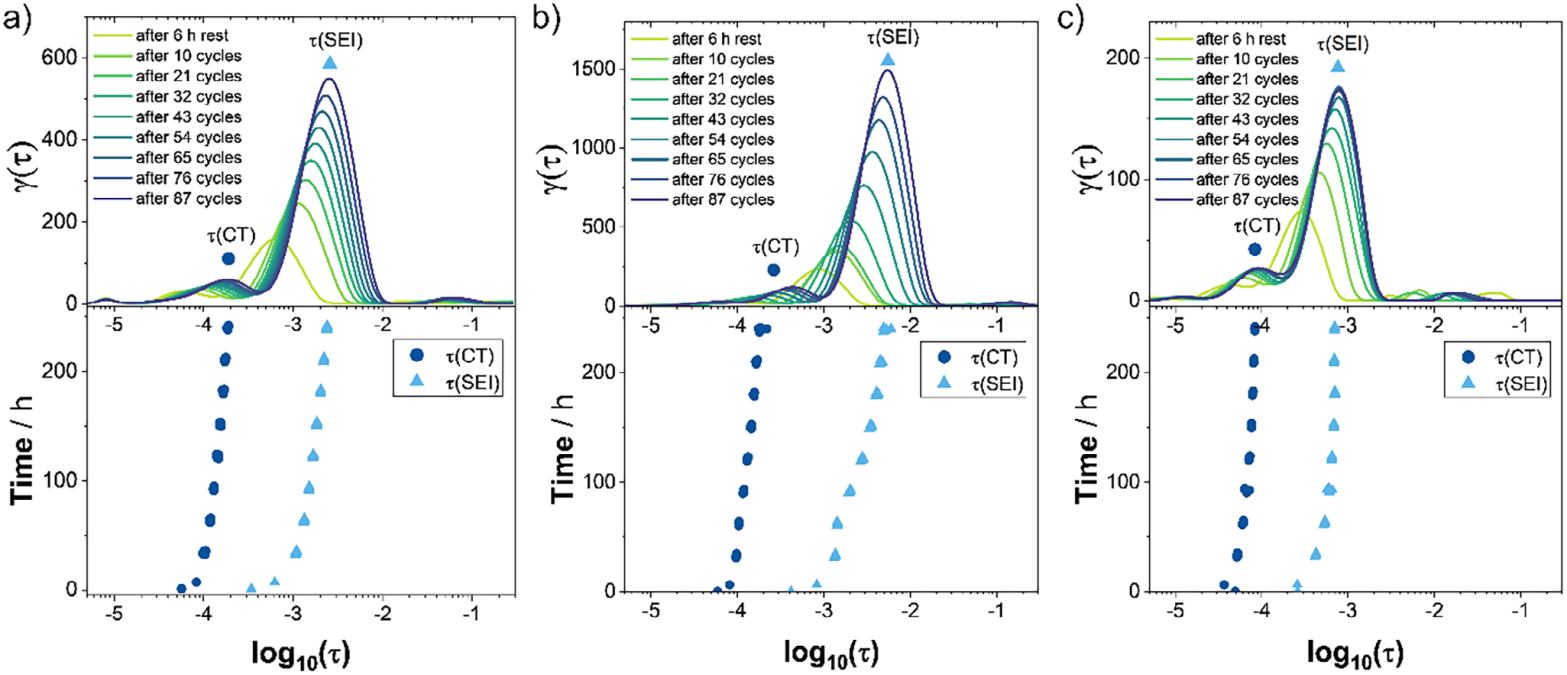
Distribution of relaxation times analysis of the EIS results acquired over the course of cycling with different polymer electrolytes. Data were recorded for a a) Li│PEO+LiTFSI│Li cell, b) Li│PEO+TEGDME+LiTFSI│Li cell, and c) Li│PEO+TEGDME+LiFTFSI│Li cell (top panels). Evolution of the time constants *τ* calculated from (R*CPE)^(1/n)^ using the values obtained from conventional equivalent circuit model fitting of the respective EIS spectra with the model circuit R‐(R)(CPE)‐(R)(CPE)‐W (bottom panels).

The EIS data obtained for these two modified polymer electrolytes upon continuous plating and stripping were also analyzed by DRT and compared with the PEO+LiTFSI baseline system. The results are shown in **Figure** **5**. As observed in Figure 3 for PEO+LiTFSI, there are two main peaks occurring in the DRT spectrum. Based on the cycling experiments (Figure 4), these can now be assigned to physicochemical processes: The peak with the smaller area (integrated peak area in DRT = resistance value) corresponds to the charge transfer resistance and the larger peak corresponds to the SEI resistance (compare also ref. [44]). In the case of PEO+LiTFSI, the peak area of the SEI (at ≈10^−3^ s) increases continuously upon cycling (Figure 5a, compare Figure 4c,d). For PEO+TEGDME+LiTFSI (Figure 5b, compare Figure 4e), the SEI‐related DRT peak shows a more pronounced increase than without TEGDME, and in the case of PEO+TEGDME+LiFTFSI (Figure 5c, compare Figure 4f), the peak size shows limited growth behavior because *R*_SEI_ stabilizes after around 150 h (i.e., 50 cycles; please note that the *y*‑axis scales differ in Figure 5).

Beyond the agreement between the fitting results from conventional equivalent circuit fitting and AutoEIS (Figure 4c,d), we evaluated the compatibility between the conventional EIS fitting and the DRT analysis by comparing the time constants. The DRT time constants can be extracted from the peak position on the *x*‐axis, the time constants from the conventional equivalent circuit model fitting can be calculated by (R*CPE)^1/^ *^n^*. Now, when comparing the time constants from DRT in the upper panel of Figure 5a–c with the time constants calculated from the conventional equivalent circuit model fitting in the lower panel of Figure 5a–c, a good agreement is observed. For all three materials, a similar evolution of the time constants for *τ*(charge transfer) and *τ*(SEI) was observed from DRT and conventional equivalent circuit fitting. Similar time constants as the values reported herein, with *τ*(charge transfer) around 10^−4^ s and *τ*(SEI) around 10^−3^ s can be found in previous studies.^[^ ^57^, ^58^ ^]^ Schmidt et al.,^[^ ^57^ ^]^ who did not distinguish between charge transfer and SEI at the anode side, reported a time constant of 5 × 10^−3^ s at 40 °C with lithium metal in a liquid electrolyte (1 m LiClO_4_ in ethylene carbonate and ethyl methyl carbonate), which is in the same range as the values obtained herein. In addition, Chen et al.^[^ ^58^ ^]^ also determined time constants between 10^−2^ and 10^−3^ s for the charge transfer and the SEI at the interface with lithium metal.

With the charge transfer and SEI‐related impedance contributions now quantitatively resolved and validated via multiple methods, the charge transfer resistance *R*_ct_ can be used to extract the exchange current density from the EIS data, as elaborated in the following section, complemented by an alternative approach via CV and, thus, enabling the cross‐validation of these two techniques.

### 2.3 Determining the Exchange Current Density by CV as an Alternative Approach to EIS

With the suitable equivalent circuit model for our Li│PEO│Li system established and confirmed (Section 2.1), and the charge transfer resistance identified (Section 2.2), the necessary information is now available to determine the exchange current density by EIS. The charge transfer resistance can be obtained from a suitable equivalent circuit model or DRT fitting and easily transformed into the exchange current density via Equation (1). Since the *R*_ct_ values that were extracted during the cycling experiment could be influenced by surface area changes, *j*_0_ was determined by EIS at OCV after a sufficient rest time to enable cell equilibration (compare Figure S4, Supporting Information, and the related text in the Supporting information). For the Li│PEO+LiTFSI│Li cells prepared at MIT, measured at 40 °C after a 2‐h heat treatment at 80 °C, *j*_0_ was determined to be 0.34 ± 0.08 mA cm^−2^.

**Figure 6:**
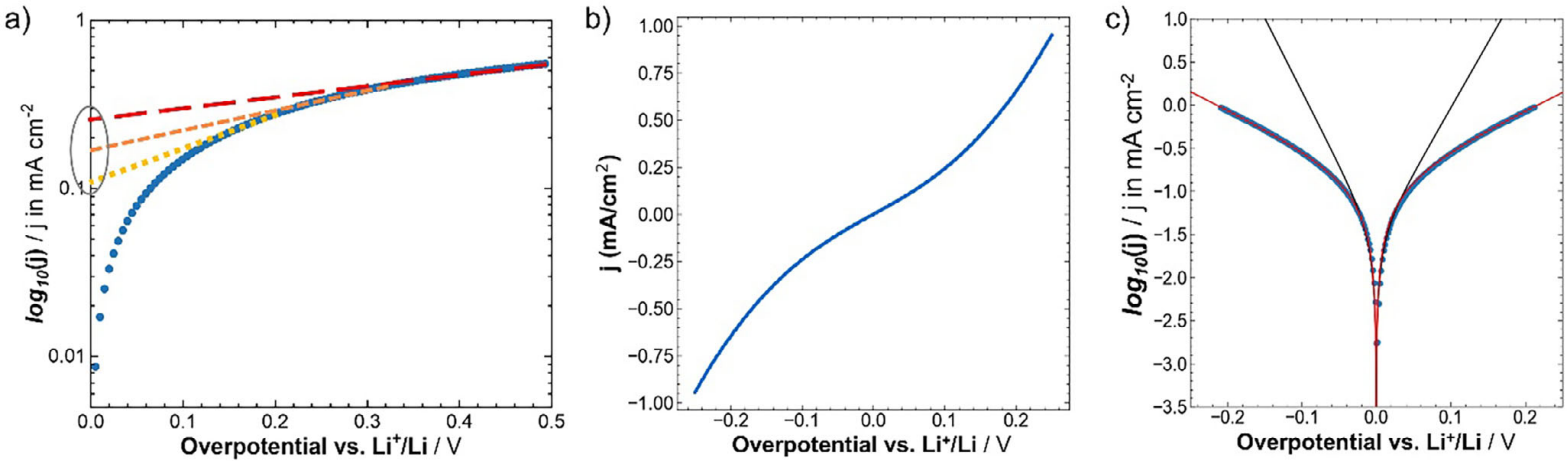
Linear sweep (LSV) and cyclic voltammetry (CV) data analysis. a) Tafel plot of LSV data of a Li│PEO+LiTFSI│Li cell recorded at 40 °C after 24 h of rest. The lines in yellow, orange, and red illustrate “linear fits” performed in different potential regions, leading to different values for the exchange current density, as highlighted by the gray ellipse. b) Forward and backward scan averaged CV data, recorded for Li│PEO+LiTFSI│Li cells at 40 °C after a 2‐h heat treatment at 80 °C at a scan rate of 100 mV s^−1^. c) Tafel plot of the CV data in (b) depicted in blue; in black: fit to the Butler–Volmer kinetic model; in red: fit to the modified Butler–Volmer kinetic model including the film resistance term.

To validate this exchange current density value determined by EIS, a second approach to determine the exchange current density was performed. As mentioned in the introduction, a standard method of electrochemists for determining the exchange current density is to perform linear sweep voltammetry (LSV) or cyclic voltammetry (CV). In this case, the scanned potential is plotted versus the current response on a logarithmic scale—the Tafel plot. From the obtained Tafel plot, the exchange current density is then typically determined from a linear regression in the “linear region,” yielding the *y*‐axis intercept, *j*_0_. This standard procedure is problematic due to several reasons. For one, identifying the linear region in the Tafel plot by eye might seem trivial; however, it can lead to critically wrong results.^[^ ^17^ ^]^ **Figure** **6**a displays three “possible” linear regressions depending on the potential range chosen for the linear fit, i.e., between 0 and 0.2 V versus Li^+^/Li (yellow dotted lined), between 0 and 0.33 V versus Li^+^/Li (dashed orange line), or between 0 and 0.5 V versus Li^+^/Li (dashed red line). These different linear regressions would obviously result in different *y*‑axis intercepts, meaning different exchange current density values. A slight curvature of the data makes a meaningful linear regression impossible, and slight deviations in the linear regression have a significant impact on the determined exchange current density values.^[^ ^17^ ^]^ Hence, instead of performing a linear regression that can lead to wrong results, in this study (forward and back scan averaged) CV data (see Figure 6b) were directly fitted to the Butler–Volmer equation (Equation (3))—a well‐known macroscopic empirical model for interfacial redox reaction kinetics for a heterogeneous one‐step, one‐electron process when the charge transfer is the rate limiting step.^[^ ^13^ ^]^

(3) $$j=j_{0}\left(\exp\left(-\frac{\alpha zF}{RT}\eta \right)-\exp\left(\frac{\left(1-\alpha \right)zF}{RT}\eta \right)\right)$$

Apart from the exchange current density *j*_0_, the equation contains the transfer coefficient *α* that represents the energy barrier symmetry, the number of transferred electrons *z*, the Faraday constant *F*, the ideal gas constant *R*, the temperature *T* in K, and the surface overpotential *η*.

As evident from Figure 6c, the Butler–Volmer model (in black) does not represent the *iR*‐corrected experimental data (in blue) well. Our findings from EIS indicate that there is a large resistive contribution from the SEI layer (compare Figure 4c,d and Figure 5a) in a Li│PEO│Li cell. This SEI resistance is not yet accounted for by the *iR*‐correction when performing the Butler–Volmer fit. Therefore, an additional film resistance term was introduced into the Butler–Volmer equation, resulting in Equation (4):

(4) $$\begin{array}{ccc} j & = & j_{0}\left(\;\exp\left(\;-\frac{\alpha zF}{RT}\left(\eta_{total}-jR_{film}\right)\right)\;-\;\exp\left(\;\frac{\left(1-\alpha \right)zF}{RT}(\eta_{total}\;-jR_{film)}\;\right)\;\right)\\ & = & j_{0}\left(\exp\left(-\frac{\alpha zF}{RT}\eta_{surface}\right)-\exp\left(\frac{\left(1-\alpha \right)zF}{RT}\eta_{surface}\right)\right) \end{array}$$

This idea and its implementation were first presented in a previous study.^[^ ^45^ ^]^ Based on some additional practical considerations regarding fitting, as outlined in the Supporting Information, the Tafel data were fitted to Equation (S2) (Supporting Information). Figure 6c shows that this modified Butler–Volmer equation yields a very good fit between the model (in red) and the experimental data (in blue). The resulting per‐interface values for *j*_0_ and *R*_film_ averaged over several Li│PEO+LiTFSI│Li cells, measured at MIT at 40 °C, are *j*_0_ = 0.21 ± 0.06 mA cm^−2^ and *R*_film_ = 343 ± 35 Ω cm^2^.

To determine these values, we used dynamic CV in a Li││Li cell geometry, because it is a simple and fast method—and in this case preferable over the static method that requires much longer measurement times (e.g., 24 h instead of 8 min),^[^ ^59^ ^]^ because within 24 h, the SEI resistance is growing non‐negligibly (compare Figure S5b, Supporting Information), which influences the Tafel slope, thereby distorting the results for *j*_0_. The Li││Li cell geometry was used because incorporating the commonly used ultra‐/microelectrodes^[^ ^18^, ^60^, ^61^ ^]^ into polymer electrolytes is experimentally very challenging. Moreover, the large SEI contribution would still have to be accounted for. The herein presented experimental and fitting method that considers the film resistance, therefore, presents a real improvement over the state‐of‐the‐art CV *j*_0_ analysis method, while encompassing a good trade‐off between experimental feasibility and data reliability.

**Figure 7:**
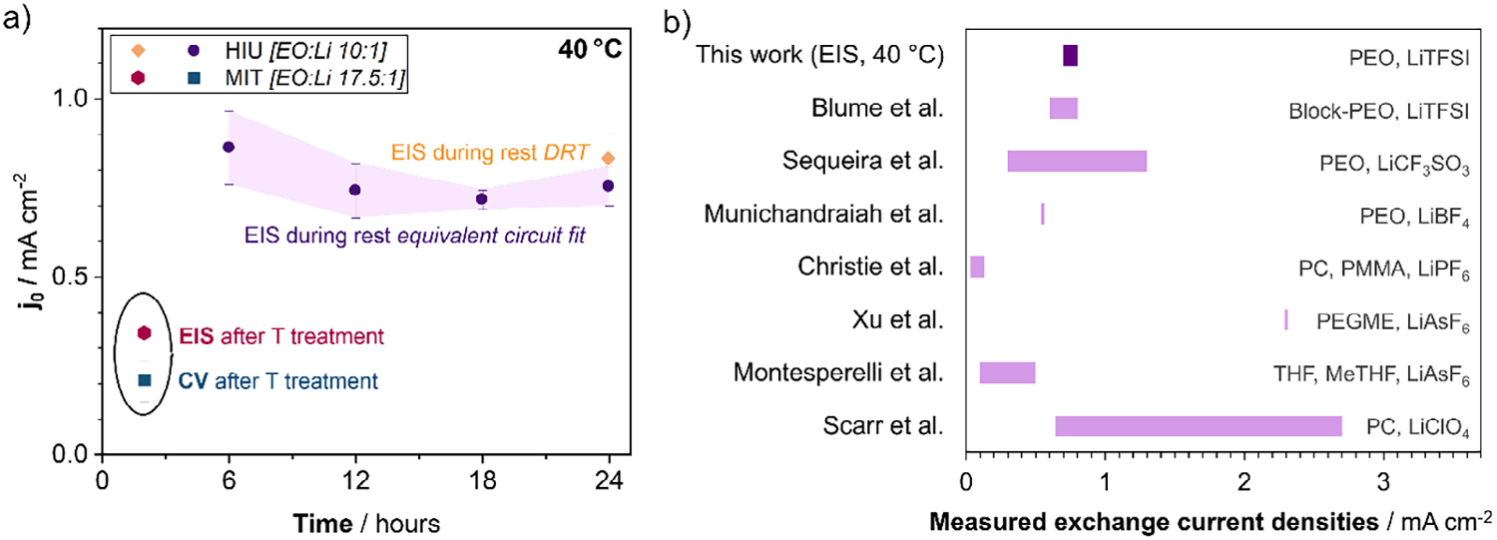
Comparison of the different exchange current density values obtained in this study and values reported earlier in the literature. a) Exchange current density values obtained herein via different methods in the two labs for Li│PEO+LiTFSI│Li cells at 40 °C. All *j*_0_ values were normalized to represent one half‐cell. In purple and orange: exchange current density extracted from EIS spectra during 24 h rest by equivalent circuit model fitting (purple balls) or by DRT fitting (orange rhombus) at HIU. In pink and green: exchange current density values determined at MIT by EIS (pink hexagon) and by CV (green square), both after a preceding 2‐h heat treatment at 80 °C. b) Compilation of exchange current density values reported in the literature and comparison with the results obtained herein.

To be able to assess the reliability of the *j*_0_ determination methods, the *j*_0_ values determined by the CV fitting method were compared to the *j*_0_ values obtained for the same system (i.e., Li│PEO+LiTFSI│Li, studied at MIT and measured at 40 °C after a 2‐h heat treatment at 80 °C) by EIS. The per‐interface exchange current density value from the CV data (0.21 ± 0.06 mA cm^−2^) agrees well with the *j*_0_ from EIS (0.34 ± 0.08 mA cm^−2^), as shown in **Figure** **7**a. Moreover, the SEI resistance that is a free fitting parameter in both analyses matches well with 440 ± 80 Ω cm^2^ (from EIS) and 343 ± 36 Ω cm^2^ (from CV). The agreement between the values determined by these two very different methodological approaches confirms the validity of both approaches, especially with regard to the large discrepancy of exchange current density values reported so far in the literature.^[^ ^14^ ^]^

### 2.4 Method Validation and Discussion

The comparison between the two methods, i.e., EIS and modified CV fitting, applied for determining *j*_0_ in the previous paragraph has already shown the feasibility and reliability of the herein proposed EIS analysis for determining the exchange current density in the case of PEO‐based electrolytes in lithium‐metal battery cells. The EIS methodology was further validated by applying the same analysis in a different lab (HIU)—to start with, for the standard composition used at HIU, i.e., EO:Li 10:1 (see Figure 7a). The exchange current density determined by equivalent circuit model fitting of an EIS spectrum (acquired after 24 h at 40 °C) was 0.75 ± 0.05 mA cm^−2^. This is in close agreement to the exchange current density extracted from the respective DRT analysis (0.83 ± 0.07 mA cm^−2^), again showing a good agreement between the equivalent circuit model fitting and the DRT analysis.

While these values differ from the 0.34 ± 0.08 mA cm^−2^ (from EIS) and 0.21 ± 0.06 mA cm^−2^ (from CV) that were determined at MIT (cf. Figure 7a), some difference is expected due to the different experimental conditions, i.e., the different salt content, differences concerning the film preparation (wet film casting vs dry hot‐pressing with crosslinking), and, in particular, the post‐assembly 2 h heat‐treatment performed at MIT; the latter being presumably the most influential factor. In fact, it is known for PEO‐based electrolytes that the thermal history influences the results obtained at lower temperatures (if *T*_past_ > *T*_current_).^[^ ^62^ ^]^ Moreover, as mentioned by Fauteaux,^[^ ^32^ ^]^ such a high‐temperature treatment can influence the active contact area between the polymer electrolyte and the lithium metal. Due to the formation of a passivating film, the electrochemically active surface area can get reduced,^[^ ^32^ ^]^ which increases the measured *R*_ct_ that is only normalized to the geometric cell area, not the real electrochemically active surface area. Resulting from the direct antiproportionality between *R*_ct_ and *j*_0_, a measured *R*_ct_ increase (due to a smaller active surface area) results in a lower *j*_0_. Looking at Figure S5b (Supporting Information) at 40 °C and Figure S5c (Supporting Information) at 80 °C, it can be seen that the passivating SEI is formed faster at higher temperature, as evident from the different *R*_SEI_(*t*). Therefore, the high‐temperature treatment is expected to have a noticeable influence on the active surface area through passivation and, thereby, also on the measured *j*_0_. To assess this experimentally, similar polymer electrolytes with an EO:Li ratio of 17.5:1 like at MIT were prepared at HIU and tested under essentially the same experimental conditions, i.e., at 40 °C after a 2 h heat‐treatment step at 80 °C. And, indeed, applying these experimental conditions, a similarly low *j*_0_ of around 0.3 mA cm^−2^ was determined after heat treatment (see Figure S6, Supporting Information, compare with Figure 7a for the results obtained at MIT), further confirming the validity of the herein presented experimental approach.

The comparison of the exchange current density values obtained herein to those reported in the literature, however, appears very challenging owing to (i) the lack of reliable data for polymer electrolytes, (ii) the commonly (very) different experimental conditions, and (iii) methodological differences. Nevertheless, Figure 7b provides a direct comparison for the sake of comprehensiveness. Blume et al.,^[^ ^20^ ^]^ for instance, have investigated a very similar system as in this study, i.e., a polystyrene‐*block*‐PEO polymer electrolyte with LiTFSI as the conducting salt and an EO:Li ratio of 10, just like the system studied at HIU. They used a millisecond current pulse technique, so that any potential concentration polarization can be neglected. *j*_0_ was determined to be 0.7 ± 0.1 mA cm^−2^ at 40 °C, which agrees very well with our findings.^[^ ^20^ ^]^ Sequeira et al.^[^ ^63^ ^]^ and Munichandraiah et al.^[^ ^64^ ^]^ also determined exchange current density values for PEO‐based polymer electrolytes, but at elevated temperatures. Micropolarization measurements yielded exchange current densities between 0.3 and 1.3 mA cm^−2^, depending on the applied pressure, with PEO+LiCF_3_SO_3_ at 100 °C.^[^ ^63^ ^]^ Galvanostatic linear polarization yielded a *j*_0_ of 0.55 mA cm^−2^ with PEO+LiBF_4_ at 80 °C.^[^ ^64^ ^]^ While these values are in a similar range compared to the ones determined in this study by EIS and CV (compare Figure 7), larger values would be expected at higher temperatures.^[^ ^45^ ^]^ In both studies, in fact, the authors discussed a potential impact of a passive SEI layer, meaning that the real *j_0_* values should be higher because the passive layer film formation leads to lower *j*_0_ values.^[^ ^63^, ^64^ ^]^ When determining the exchange current density for propylene carbonate‐poly(methyl methacrylate) gel‐type electrolytes with LiPF_6_ as conducting salt and a nickel microelectrode by non‐linear least squares fitting the reverse sweep of voltammograms to the Butler‐Volmer equation, Christie and Vincent^[^ ^65^ ^]^ also did not take into account the influence of a surface film. This, and the influence of the lower temperature (room temperature) might explain why the therein reported *j*_0_ of 0.13 mA cm^−2^ is smaller than the values reported herein. In a study with a liquid (though chemically similar) electrolyte (poly(ethylene glycol) methacrylate+LiAsF_6_), Xu and Farrington^[^ ^60^ ^]^ employed nickel microelectrodes to determine the exchange current density via potential sweep and potential step experiments, yielding a value of 2.3 mA cm^−2^. Although this value is larger than ours, it fits well with our data considering that it was obtained in a liquid system at a higher temperature (50 °C), for which a higher *j*_0_ is expected. Besides, exchange current densities of 0.1–0.5 mA cm^−2^ have been reported for 1.5 m LiAsF_6_ in tetrahydrofuran/2‐methyltetrahydrofuran using EIS,^[^ ^66^ ^]^ and between 0.64 and 2.7 mA cm^−2^ for 1 m LiClO_4_ in propylene carbonate using a current interruption and pulse method, although the latter values appeared to depend largely on the method and the water content in the glovebox.^[^ ^67^ ^]^ While these values were obtained for liquid electrolyte systems, for which a higher *j*_0_ is expected, and were acquired at room temperature instead of 40 °C, for which a lower *j_0_* would be expected, they appear still comparable, thus, further corroborating the validity of the approach proposed herein.

## 3 Conclusion

In this study, we have provided an easily implementable toolbox for determining the exchange current density values of lithium‐metal batteries employing polymer electrolytes. It has been shown that EIS is a readily applicable method for determining the exchange current density in lithium‐metal cells, as exemplified for PEO‐based polymer electrolytes. The application of various complementary methods, including conventional equivalent circuit model fitting, Bayesian inference analysis (AutoEIS), DRT, and an extensive cycling study allows for the identification of the most suitable equivalent circuit model for Li│PEO│Li cells and to clarify the distinct contributions of the charge transfer resistance and the SEI resistance—a distinction often overlooked in literature, even though being crucial for correctly determining the exchange current density. To validate *j*_0_ as determined by EIS, a modified Butler–Volmer fitting of CV data, incorporating a fitting parameter for the SEI resistance, was performed, providing consistent *j*_0_ values that align well with the EIS results. The comparison of data obtained in two different labs confirms the reliability of the proposed methodology. Hence, the herein proposed method for determining *j*_0_, proven suitable for PEO‐based electrolytes, is anticipated to advance the understanding of interfacial kinetics at Li│polymer interfaces and may help to standardize future measurements, thus, helping to enhance the comparability of future studies in this field.

## 4 Experimental Section

Generally, since experiments were conducted in two different labs, i.e., at HIU and at MIT, the experimental procedures varied slightly, as described in the following sections.

### Polymer Electrolyte Fabrication

At HIU, the polymer electrolytes were prepared via an already established solvent‐free method.^[^ ^68^, ^69^ ^]^ Polyethylene oxide (PEO, *M*_W_ = 4 Mio. g mol^−1^, Sigma Aldrich), LiTFSI as conducting salt (3 m), and benzophenone (purity ≥ 99%, Merck) were mixed in a molar ratio of [100:10:1] for [EO: LiTFSI: benzophenone]. For the polymer electrolytes containing tetraethylene glycol dimethyl ether (TEGDME), also referred to as tetraglyme, serving as liquid plasticizing agent, a 50:50 wt% mixture of PEO and tetraglyme was used instead of pure PEO. LiFTFSI as alternative conducting salt was purchased from Provisco CS. All components were dried in a Büchi vacuum oven prior to use: PEO at 50 °C for 48 h, LiTFSI at 120 °C for 24 h, LiFTFSI at 80 °C for 48 h, and benzophenone at room temperature for 24 h. After mixing the dried powders until a chewing gum‐like consistency was obtained, the mixture was vacuum‐sealed in a pouch bag and annealed overnight at 100 °C in an annealing oven (Binder E 28). Polymer membranes with a thickness of ≈120 µm were obtained by hot‐pressing at 90 °C up to a maximum pressure of 20 bar (Polystat 200 T, Servitec). The obtained films were crosslinked in a UV‐light chamber (UVACUBE 100, hönle uv technology) from both sides for 6 min each to prevent crystallization of the generally amorphous material and to improve the mechanical stability. All steps were performed in a dry room (dew point ≈ −70 °C ≡ < 2 ppm H_2_O).

At MIT, the polymer electrolytes were prepared by combining PEO (*M*_W_ = 600 000 g mol^−1^, Sigma‐Aldrich) with LiTFSI (Sigma‐Aldrich) in an argon‐filled glove box using a [17.5:1] molar ratio of EO to LiTFSI. These solid powders were dissolved in acetonitrile to yield a 2% by weight solution of PEO. The solution was left to stir at ambient temperature until all materials were completely dissolved. Then, 3 × 100 µL aliquots of the solution were drop cast onto 1 cm diameter Teflon substrates, allowing the acetonitrile to evaporate between each aliquot. The resulting films were dried under vacuum at 80 °C for 6 h.

### Symmetric Cell Fabrication

At HIU, symmetric Li│polymer│Li pouch cells (Honjo Metal Co.) with nickel current collectors were prepared under dry room conditions (with a dew point ≈ −70 °C ≡ < 2 ppm H_2_O) with the active cell area ranging between 1 and 2 cm^2^. After assembly and vacuum sealing, the pouch cells were laminated (GBC Fusion 1000L) to ensure good contact between the polymer film and the lithium‐metal electrode.

At MIT, Li│polymer│Li cells were prepared in an argon‐filled glove box (O_2_, H_2_O < 0.1 ppm) in Swagelok T‐cells using stainless steel dies as current collectors. Prior to battery assembly, lithium foils (MTI, 6 mm diameter, 150 µm thickness) were polished in the glove box using a steel spatula to remove the native oxide surface layers. To form a stable passivation layer at the Li│PEO interface, the symmetric cells were heated to 80 °C for 2 h in a muffle furnace inside the glove box, and then cooled to room temperature before further testing.

### Electrochemical Testing

At HIU, EIS was performed using a Solartron SI 1287 or VMP‐3e device (BioLogic). Measurements were conducted in a frequency range from 1 MHz to 100 mHz with an excitation amplitude of 10 mV, following different rest times (e.g., 6 or 24 h) at 40 °C, if not stated otherwise. For the cycling experiments, constant‐current plating/stripping was performed at a current density of 0.1 mA cm^−2^ with an areal capacity of 0.1 mAh cm^−2^ per half‐cycle. After every 10 cycles, EIS was repeated following a 15‐min rest period.

At MIT, all electrochemical measurements were performed using a Biologic SP‐300 potentiostat at 40 °C, if not stated otherwise. EIS was conducted in a frequency range from 7 MHz to 1 Hz with an excitation amplitude of 10 mV. Cyclic voltammetry (CV) was carried out using a sweep rate of 100 mV s^−1^. All CV data obtained for the symmetric cells are reported as the cell voltage divided by two in order to represent the voltage of the working electrode versus Li^+^/Li.

### Data Analysis

At HIU, the software RelaxIS3 (rhd instruments) was used for conventional equivalent circuit model fitting and DRT analysis. The specific equivalent circuit models used are specified in the results section. For the DRT transformation of the EIS spectra, the data between 1 MHz and 5 Hz were used and the low‐frequency diffusion part was removed.^[^ ^70^ ^]^ The λ regularization parameter was carefully chosen between 0.0001 and 0.001 following ref. [42] by lowering λ until the sum of square residuals between the experimental data and the reproduced spectrum from DRT reached a stable value. Since λ influences the number of peaks and their shape, a suitable balance between accurate data representation (risk of underfitting) and avoiding artificial peaks that arise from measurement uncertainty (overfitting) had to be found. Hence, a careful pre‐evaluation of λ was eminent to obtain meaningful results.^[^ ^24^, ^42^, ^71^ ^]^ The DRT peaks were fitted with a Gaussian function to determine the peak area representing the respective resistance value. For the EIS data collected at MIT, an independent DRT analysis was performed using the DRT.jl package in Julia with a λ regularization parameter of 0.01.^[^ ^72^ ^]^

Bayesian inference analysis was performed with the AutoEIS tool recently developed by Zhang et al.^[^ ^40^ ^]^ Here, different plausible equivalent circuit models with prior default values for each circuit element were inserted as inputs for the AutoEIS python code. Subsequently, via Bayesian inference statistics, posterior probability distributions were generated for the values of each circuit element, as were goodness of fit metrics that enable a statistical determination of the most appropriate of the different equivalent circuit models.

The CV data were fit to a Butler–Volmer model of interfacial charge transfer kinetics that includes parameters for the exchange current density *j*_0_, the charge transfer coefficient *α*, and the film resistance of the SEI, *R*_film_ (see Equation (4)). Forward and reverse CV sweeps were averaged to generate a single curve for the Butler–Volmer fitting. Prior to fitting, all CV data were *iR*‐corrected to account for the voltage drop associated with the resistance of the bulk ion transport as determined from EIS measurements.

## Conflict of Interest

The authors declare no conflict of interest.

## Author Contributions

K.G. and B.T. conducted the experiments and analysis in a collaborative effort under supervision of D.B. and Y.S.‐H., respectively. K.G. prepared the first draft of the manuscript based on experiments performed by K.G. and B.T. following fruitful discussions with B.T., S.P., and D.B. B.T. performed the statistical analysis with AutoEIS and the CV experiments, and compiled the corresponding figures. All authors revised and edited the draft, making valuable contributions to improve the manuscript.

## Supporting information



Supporting Information

## Data Availability

The data that support the findings of this study are available from the corresponding author upon reasonable request.
