# Supplementary material for: Determination of the Exchange Current Density at Lithium │ Polymer Electrolyte Interfaces
Source: Adv Sci (Weinh). 2025 Nov 27;13(8):e14492. doi: 10.1002/advs.202514492 (PMC12884815; doi:10.1002/advs.202514492)
Supplement: Supplementary file 1 — Supporting Information [file ADVS-13-e14492-s001.docx]

Supporting Information

**Determination of the exchange current density at lithium│polymer electrolyte interfaces**

Katrin Geng,^a,b^ Bryce A. Tappan,^c^ Stefano Passerini,^a,b,d^ Yang Shao-Horn,^c,e,f,*^ Dominic Bresser^a,b,g,*^

1. Helmholtz Institute Ulm (HIU), 89081 Ulm, Germany
2. Karlsruhe Institute of Technology (KIT), 76131 Karlsruhe, Germany
3. Research Laboratory of Electronics, Massachusetts Institute of Technology (MIT), Cambridge, Massachusetts 02139, USA
4. Austrian Institute of Technology (AIT), Center for Transportation Technologies, 1210 Vienna, Austria
5. Department of Materials Science and Engineering, Massachusetts Institute of Technology (MIT), Cambridge, Massachusetts 02139, USA
6. Department of Mechanical Engineering, Massachusetts Institute of Technology (MIT), Cambridge, Massachusetts 02139, USA
7. Ulm University (UUlm), 89069 Ulm, Germany

*E-Mail corresponding authors: dominic.bresser@kit.edu; shaohorn@mit.edu

**
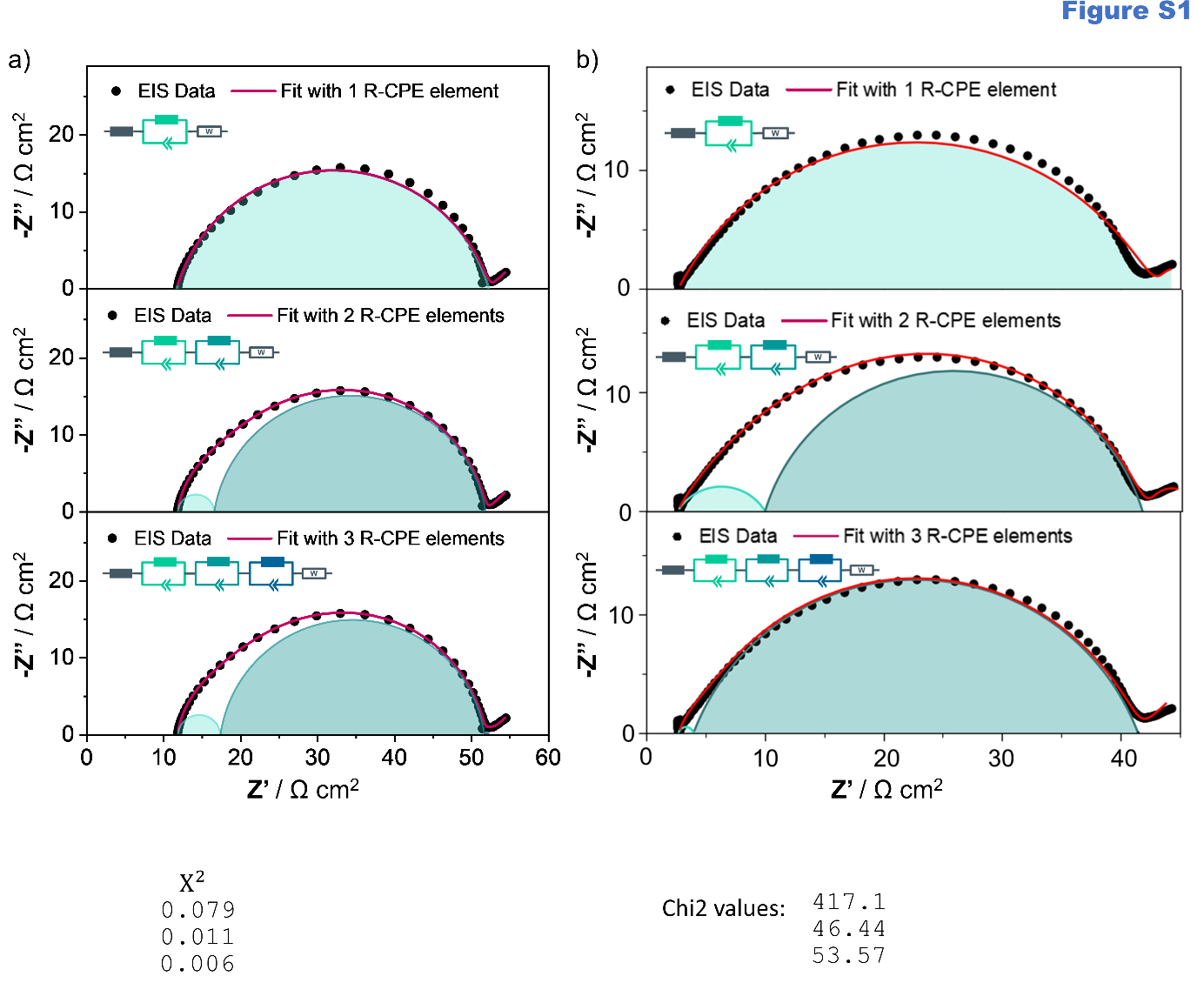
**

**Figure S1.** Fitting of EIS data recorded for Li│PEO+LiTFSI│Li cells at HIU and MIT with three different equivalent circuit models. (a) Conventional equivalent circuit model fitting of an impedance spectrum recorded for a Li│PEO+LiTFSI│Li cell at 80 °C after 6 h of rest (conducted at HIU). The equivalent circuit models used for fitting are displayed in each subgraph, the mid-frequency region (from 100 kHz to 1 Hz) was fitted with one, two, or three R-CPE elements (from top to bottom). (b) Impedance spectrum of a Li│PEO+LiTFSI│Li cell recorded at 80 °C after 2 h of rest (conducted at MIT). The equivalent circuit models used for fitting are displayed in each subgraph, the mid-frequency region (from 100 kHz to 1 Hz) was fitted with one, two, or three R-CPE elements (from top to bottom).

**Table S1.** Results of the conventional equivalent circuit model fitting of the impedance spectrum recorded for a Li│PEO+LiTFSI│Li cell. According to the bottom panel in **Figure 1**, three (R)(CPE)‑elements in series were used in the model for fitting the mid-frequency region. The impedance spectrum was recorded at 40 °C after 6 h of rest. Large fit errors >50% indicate overfitting and are highlighted in red, fit errors between 10% and 50% are highlighted in orange, and smaller fit errors in green.

| **Equivalent circuit model parameter name** | **Value** | **Fit Error** | |
| --- | --- | --- | --- |
| Resistance 1 | 135.1 | 0.5 | 0.4% |
| Resistance 2 | 40 | 200 | 500% |
| CPE Q 2 | 1E-05 | 1E-05 | 100% |
| CPE Alpha 2 | 0.8 | 0.3 | 38% |
| Resistance 3 | 80 | 60 | 75% |
| CPE Q 3 | 3E-05 | 3E-05 | 130% |
| CPE Alpha 3 | 0.8 | 0.3 | 38% |
| Resistance 4 | 230 | 160 | 70% |
| CPE Q 4 | 5E-06 | 2E-06 | 40% |
| CPE Alpha 4 | 0.9 | 0.1 | 5% |

**
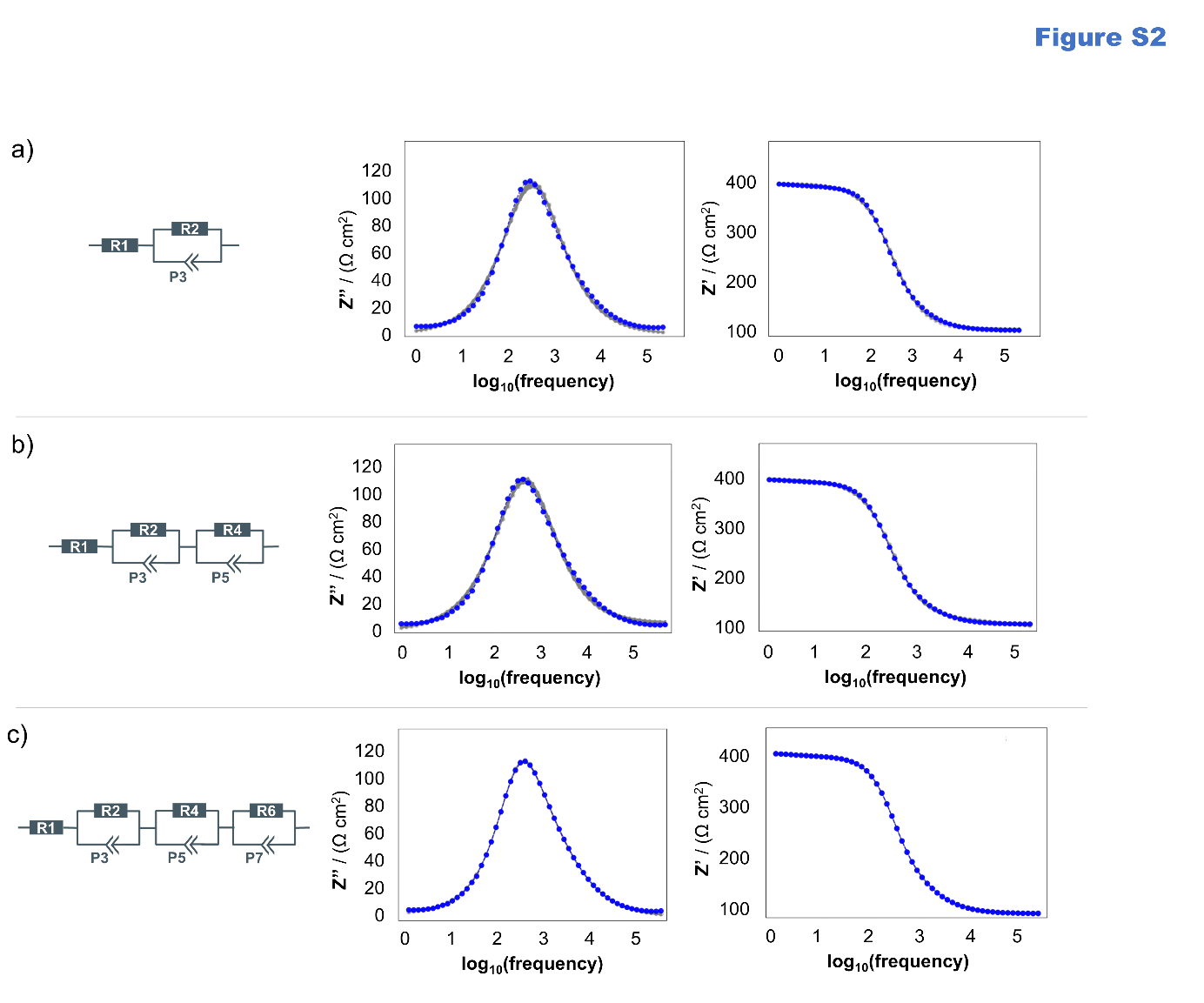
**

**Figure S2**. Bode plots from Bayesian inference analysis for three different equivalent circuit models. Results obtained using AutoEIS performed on impedance spectra recorded for Li│PEO+LiTFS│Li cells at 40 °C after 6 h of rest in the mid-frequency range (194 kHz to 1 Hz). The equivalent circuit models used are: (a) R-(R)(CPE), (b) R-(R)(CPE)-(R)(CPE), and (c) R-(R)(CPE)-(R)(CPE)-(R)(CPE). For each of these, predictive Bode plots are shown for the frequency dependent imaginary part (left) and the real part (right) of the impedance.

**
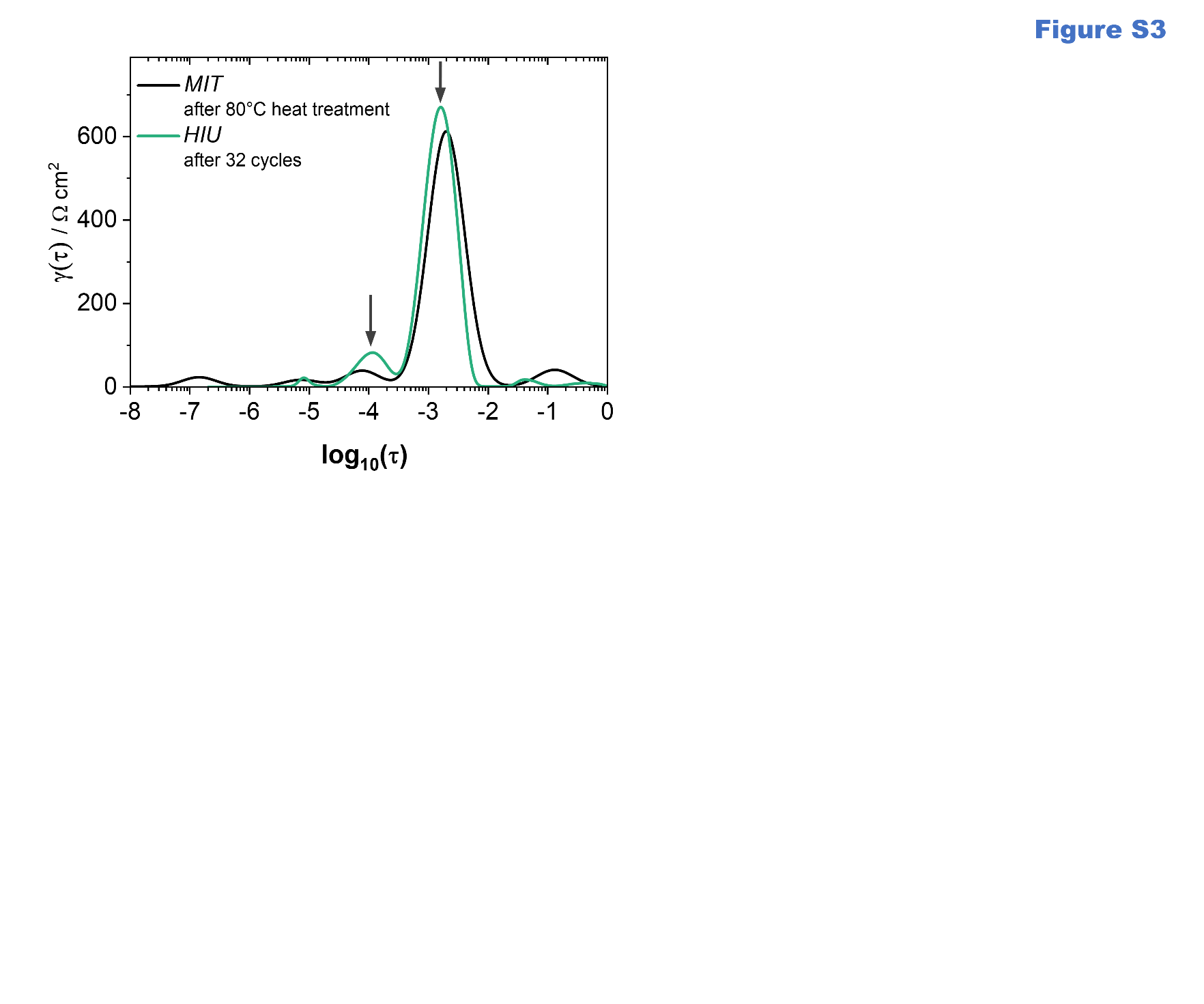
**

**Figure S3.** Comparison of the distribution of relaxation times (DRT) analyses of impedance spectra obtained at HIU and MIT. Electrochemical impedance spectroscopy on Li│PEO+LiTFSI│Li cells was conducted at HIU (in petrol; after 32 plating/stripping cycles at 0.1 mA cm^-2^ with an areal capacity of 0.1 mAh cm^-2^ per half-cycle) and MIT (in black; after a preceding 2-hour heat treatment at 80 °C); both DRT analyses exhibit two main peaks at around 10^-4^ and 10^‑3^ s.

**

**

**Figure S4.** Exchange current density values acquired by EIS during cycling. EIS recorded for Li│PEO+LiTFSI│Li cells at 40 °C during plating/stripping with a current density of 0.1 mA cm^-2^ and an areal capacity of 0.1 mAh cm^-2^ per half-cycle (experiments conducted at HIU). The *j_0_* values were normalized to represent one half-cell.

The exchange current density values determined by EIS at HIU during the cycling experiment (compare **Figures 4** and **5**) are displayed in **Figure S4**. It is observed that, especially after the onset of plating/stripping, the measured exchange current density values are decreasing remarkably and continue to do so over the course of continued cycling. In fact, an increase of charge transfer resistance (that corresponds to a decrease of exchange current density (compare **Equation** **(1)**) was also observed by Montesperelli *et al.*^[1]^ during cycling, who have observed an increase of *R_ct_* from 1 Ω cm^2^ to 30 Ω cm^2^ over 450 cycles (using a liquid electrolyte). While the surface area is probably changing during cycling, which renders the normalization to the surface area challenging, the cycling experiment can give valuable insights into the total *R_ct_*, rendering it a powerful tool to distinguish between charge transfer and SEI resistance. After having performed the cycling experiment as a means to assign the resistances to physicochemical processes and to gain information on the system under investigation, the exchange current density can be determined with high accuracy at OCV conditions.

To determine the exchange current density by EIS at OCV, especially if no high-temperature treatment is conducted (as in the case for the experiments carried out at HIU), a certain rest period should be applied, since a freshly prepared cell requires some time to equilibrate at OCV at a given temperature (compare *R_SEI_(t)* in **Figures S5b-c**). While the “interphase impedance” (i.e., the large semicircle-type feature in the EIS spectrum containing contributions from the charge transfer and the SEI impedance) might not be stabilizing over a period of days due to SEI growth (compare **Figure S5a** and Ref.^[2]^), the values for the bulk and the charge transfer resistance stabilize after around 6 h in the case of the Li│PEO+LiTFSI│Li system at 40 °C (see **Figure S5b** and **Figure 7a**, purple circles). Therefore, in this case, a rest time of 24 h prior to EIS appears appropriate for determining the exchange current density for such a system.

**Supporting Information to the modified Butler-Volmer model** (presented in Ref. ^[3]^)

After incorporating the overpotential $jR_{film}$ in the Butler-Volmer equation (compare **Equation (3)** and **Equation (4)**), the current appears on both sides of the equation. To enable fitting, in other words, to circumvent the challenge of having the current density $j$ on both sides of the equation, $V_{drop\_coefficient}$ was introduced, representing the overpotential drop due to the SEI film resistance as a parameter between 0 and 1.

With **Equation (S1)**

| $\eta_{surface}=\eta_{total}-jR_{film}= \eta_{total}*V_{drop\_ coefficient}$ | **(S1)** |
| --- | --- |

the modified Butler-Volmer equation writes as follows (**Equation (S2)**):

| $j=j_{0}\left( \exp\left( -\frac{\alpha zF}{RT}{(\eta}_{total}*V_{drop\_coefficient}) \right)-\exp\left( \frac{\left( 1-\alpha\right)zF}{RT}{(\eta}_{total}*V_{drop\_coefficient} \right) \right)$ | **(S2)** |
| --- | --- |

This formula can easily be used for fitting the data with the exchange current density *j_0_*, $\alpha$, and $V_{drop\_coefficient}$ as fitting parameters.

By rearranging **Equation (S1)**, an approximation of the film resistance *R_film_* can be determined from **Equation (S3)**:

| $R_{film}=\frac{\eta_{total}(1-V_{drop\_ coefficient})}{j}$ | **(S3)** |
| --- | --- |

with the determined $V_{drop\_coefficient}$ = 0.27, this gives *R_film_* = 343 ± 35 Ω cm^2^.

The CV fitting procedure could be improved further by accounting for mass transport limitation at high overpotentials, which is known to cause a curvature in the Tafel plot at high overpotentials due to concentration depletion.^[4]^ Therefore, for the data analysis in this study, only values up to an overpotential of 0.2 mV vs. Li^+^/Li were used for the analysis. The “actual” potential range is even smaller because the overpotential is increased by *R_SEI_*. Generally, a mass transfer correction could be performed using a rotating disc electrode by the Koutecky-Levich compensation, but aside from a rotating disc electrode, it requires a precise determination of the limiting current,^[4]^ which is very challenging for polymer electrolytes; the latter not least because of the time and temperature dependent formation of a (partially) passivating decomposition layer (SEI) that suppresses current flow.^[5]^ Another idea for mass transport correction would be using the real concentration at the interface calculated from Fick’s 2^nd^ law in the Butler-Volmer equation, which is also not trivial since one is confronted with a 2^nd^ order diffusion equation without simple solutions.


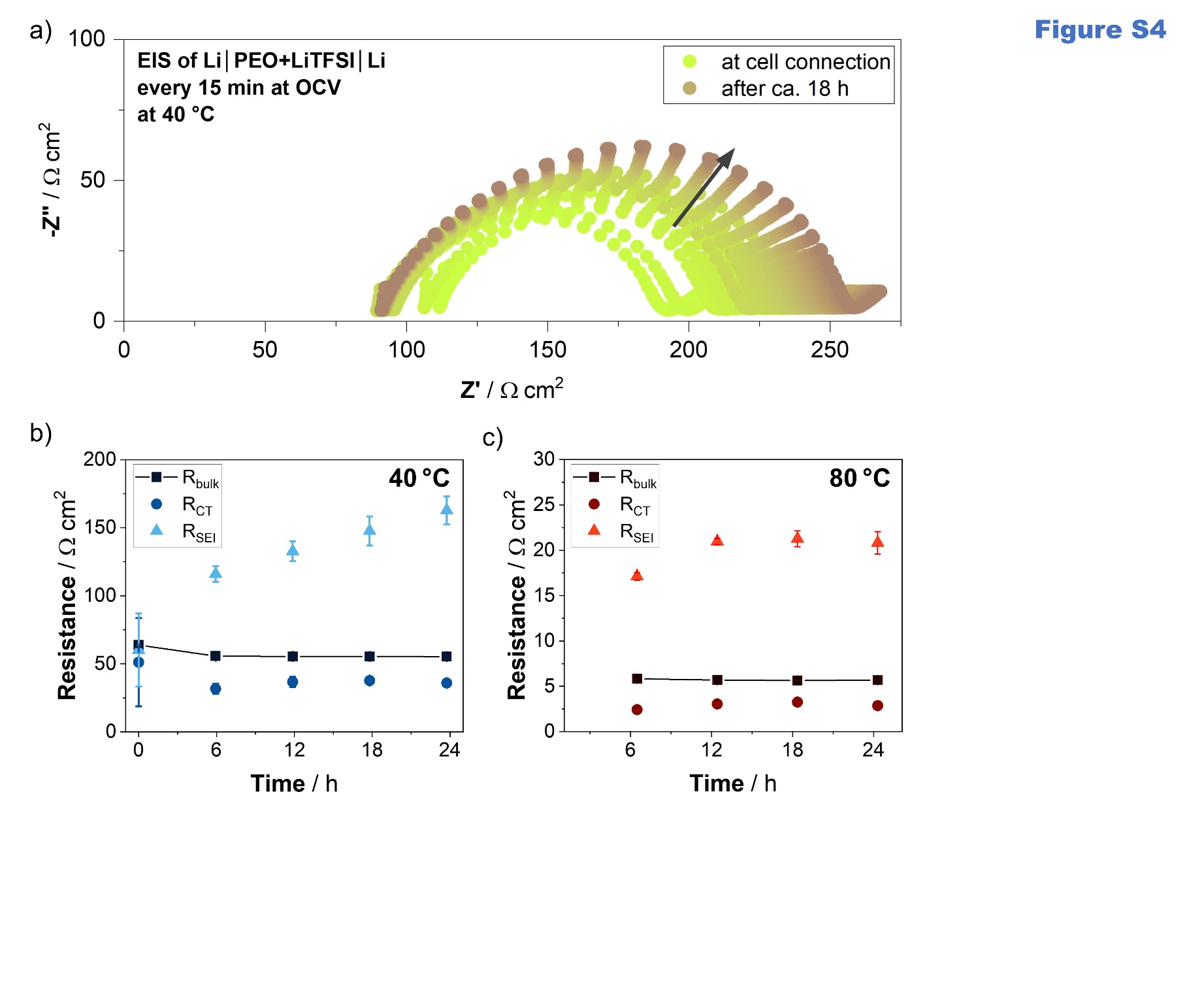


**Figure S5**. Electrochemical impedance spectroscopy analysis for Li│PEO+LiTFSI│Li cells during rest. (a) Impedance spectra recorded at 40 °C recorded from 100 mHz to 1 MHz every 15 min, showing an increase of the impedance over time. (b) Averaged resistance values obtained by fitting the impedance spectra of two cells during a rest period of 24 h at 40 °C with the equivalent circuit model displayed in **Figure 4b**. (c) Averaged resistance values obtained by fitting the impedance spectra of two cells during a rest period of 24 h at 80 °C with the equivalent circuit model displayed in **Figure 4b**.


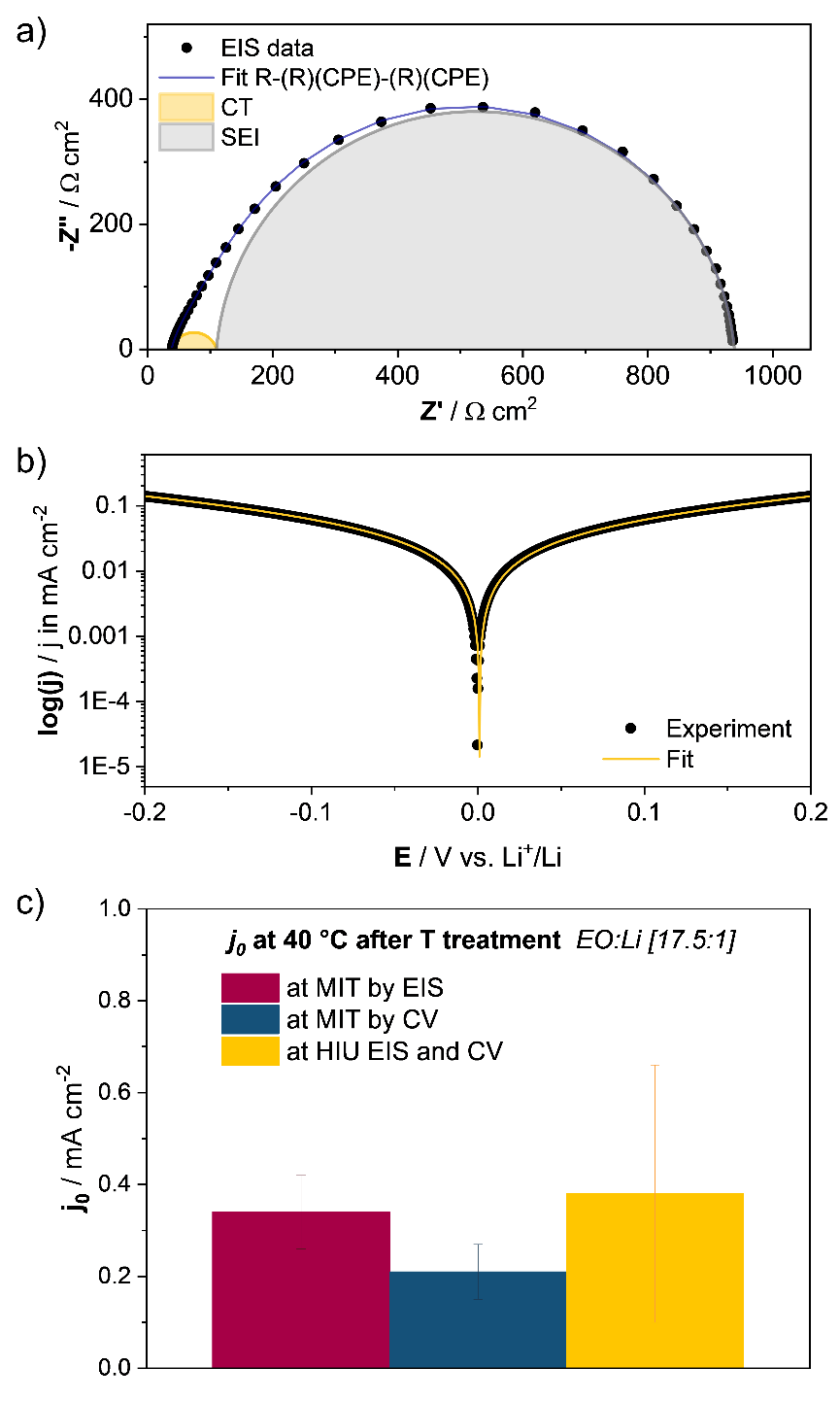


**Figure S6**. Confirmation of exchange current density results from MIT by additional experiments at HIU for a similar system with PEO:LiTFSI with an EO:Li ratio of 17.5:1 at 40 °C after 2 h of heat-treatment at 80 °C: (a) EIS fit for a Li│PEO+LiTFSI│Li cell after the heat-treatment. The Nyquist plot was fitted to the equivalent circuit model shown in the figure legend; (b) forward and backward scan averaged CV data for a Li│PEO+LiTFSI│Li cell acquired at 100 mV s^‑1^ after the heat-treatment (the CV data were fitted to the modified Butler-Volmer equation with a film resistance term, as displayed in the Tafel plot). (c) Comparison of the resulting exchange current density values determined at MIT (red and blue) and at HIU (yellow) with the similar polymer electrolyte under similar experimental condition, i.e., all at 40 °C after 2 h heat treatment at 80 °C. All *j_0_* values were normalized to represent one half-cell.

**References**

[1] G. Montesperelli, *Solid State Ionics* **1990**, *37*, 149.

[2] R. Bouchet, S. Lascaud, M. Rosso, *J. Electrochem. Soc.* **2003**, *150*, A1385.

[3] B. A. Tappan, K. Geng, D. Vivona, D. Wang, D. Mankus, A. Lytton-Jean, D. Bresser, Y. Shao-Horn, *ACS Appl. Mater. Interfaces* **2025**, *17*, 18255.

[4] P. Khadke, T. Tichter, T. Boettcher, F. Muench, W. Ensinger, C. Roth, *Sci Rep* **2021**, *11*, 8974.

[5] M. Wetjen, G.-T. Kim, M. Joost, M. Winter, S. Passerini, *Electrochimica Acta* **2013**, *87*, 779.
